# Case report: Sagliker syndrome in the patient with recurrent tertiary hyperparathyroidism due to intrathyroidal parathyroid carcinoma

**DOI:** 10.3389/fendo.2023.1292993

**Published:** 2024-01-05

**Authors:** Rustam Salimkhanov, Ekaterina Bondarenko, Anna Eremkina, Ekaterina Bibik, Ekaterina Kim, Kamila Begova, Ilya Kim, Sergey Kuznetsov, Natalia Mokrysheva

**Affiliations:** ^1^ Department of Parathyroid Pathology and Mineral Disorders, Endocrinology Research Center, Moscow, Russia; ^2^ Laboratory of Pathomorphology, Endocrinology Research Center, Moscow, Russia; ^3^ Department of Endocrine Surgery, Endocrinology Research Center, Moscow, Russia; ^4^ Administration, Endocrinology Research Center, Moscow, Russia

**Keywords:** Sagliker syndrome, parathyroid carcinoma, tertiary hyperparathyroidism, chronic renal failure, case report

## Abstract

Sagliker syndrome (SS) is an extremely rare disorder that manifests in patients with advanced chronic kidney disease (CKD) undergoing programmed hemodialysis as a renal replacement therapy. Treatment of secondary hyperparathyroidism (SHPT) in these patients is still challenging. The main clinical manifestations of SS include craniofacial and fingertip deformities, dental anomalies, gingival hyperplasia, short stature, hearing loss, neurological and psychiatric impairment. The etiology and pathogenesis of SS in patients with SHPT require further clarification. However, mutations in the *GNAS1*, *FGF23*, and *FGFR3* genes were described in some patients, suggesting a possible role of genetic predisposition to the syndrome. The preferred therapeutic approach for SS is surgery, but the volume of the operation is debated. The main surgical strategies include total, subtotal parathyroidectomy, or total parathyroidectomy with autotransplantation of the parathyroid gland (PG). Unfortunately, parathyroidectomy does not contribute to the regression of significant skeletal deformities. We present a unique clinical case of a patient with classical features of SS, recurrent tertiary hyperparathyroidism (THPT) after total parathyroidectomy due to intrathyroidal parathyroid carcinoma (PC).

## Introduction

Impaired renal function increases the risk of bone and mineral metabolism disorders. The aggressive course of SHPT due to end-stage CKD in patients with inadequate disease control contributes to the development of the most pronounced bone pathology (1). Combination of changes in bone density, mineralization, and shape with the development of marked skeletal deformities in patients with persistent renal dysfunction is commonly referred to as renal osteodystrophy or extreme manifestations of mineral and bone damage in CKD. SS is a rare form of renal osteodystrophy manifested by facial bone deformities involving the jaw apparatus, nasal and maxillary bone destruction, oral mucosa soft tissue, tooth position and structure abnormalities, short stature, changes in the fingers and toes, valgus knee and scapular deformities, gait disturbances, hearing loss, neurological and psychiatric impairments (2, 3).

SS was first described as a syndrome by the nephrologist Y. Sagliker in 2004, although patients with similar phenotypes were previously reported (4). The incidence of SS is approximately 0.5% in the population of CKD patients currently undergoing hemodialysis (1).

We describe a case report of SS in a patient with recurrent THPT after total parathyroidectomy due to intrathyroidal PC.

## Clinical case description

Patient M., a 48-years-old female, was admitted to the Department of Parathyroid Pathology and Mineral Disorders at the Endocrinology Research Centre (Moscow, Russian Federation) with severe pain in the feet during walking, lumbar region, bones and joints (hips, knees), muscle weakness, height loss, partial hearing loss.

### Past medical history

M. was diagnosed with chronic glomerulonephritis in 1984 at the age of 10 years. However, the patient declined any further testing and treatment. Since 2008, her well-being has deteriorated due to progressive CKD, resulting in worsening edema, general and muscle weakness, elevated blood pressure (up to 200/120 mm Hg), and weight loss.

Since 2009, the patient has started regular renal replacement therapy via programmed hemodialysis. Over the next four years, she developed hypercalcemia with a total calcium level of 2.7 mmol/L and an increased parathyroid hormone (PTH) to 1125 pg/mL, leading to the diagnosis of THPT (5). M. did not receive continuous treatment for THPT between 2009 and 2013; however, she was treated intermittently with cinacalcet at a dose of 30 mg/day. Despite this therapy, deterioration of renal osteodystrophy was observed. The patient presented with thoracic deformity and progressive spinal and extremity pain.

She was admitted to the Endocrinology Research Centre in 2013. Laboratory tests revealed an elevated PTH level – 3536 pg/mL (15–65), hypercalcemia – 2.8 mmol/L (2.1-2.55), and hyperphosphatemia – 1.9 mmol/L (0.74-1.52) which was consistent with the diagnosis of THPT. Neck ultrasound scan showed hyperplasia of four PGs located in typical positions (the right upper gland 1.6x1.2x0.8 cm, the right lower gland 1.7x1.0x0.7 cm, the left upper gland 3.8x2.4x1.8 cm, and the left lower gland 1.1x0.8x0.8 cm).

**Figure 1:**
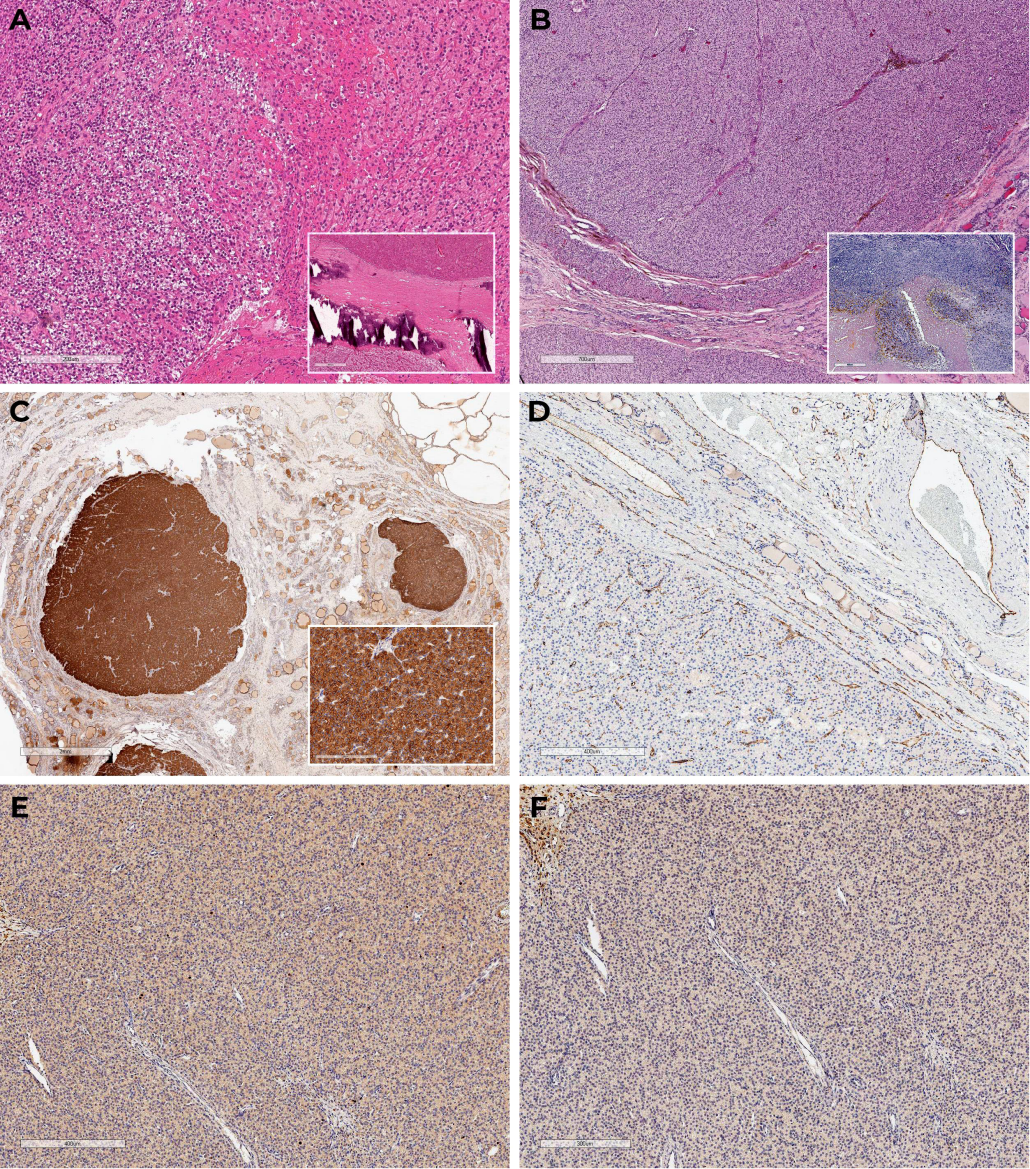
Histoscans **(A)** – parathyroid hyperplasia, material after surgery in 2013; **(B–F)** – PC, material after surgery in 2023. **(A)** Diffuse parathyroid hyperplasia (H&E); **(B)** Oxyphilic cells with areas of necrosis, fresh and old hemorrhages, tumor surrounded by a capsule with areas of invasive growth and vascular protrusions (H&E); **(C)** Diffuse expression of PTH in the tumor, negative in the surrounding tissue; **(D)** CD31 – absence of vascular invasion signs; **(E)** Ki-67 3%; **(F)** Loss-of-expression of parafibromin.

Total parathyroidectomy was conducted in 2013. During the revision the thyroid gland was not enlarged, no nodular formations were found. Four enlarged PGs were removed in their typical locations without autotransplantation. The thymus and its ligaments were not affected during the surgery. Histological examination confirmed diffuse parathyroid hyperplasia (**Figure 1A**), immunohistochemistry was not performed.

During the postoperative period, M. experienced severe hypocalcemia, with ionized calcium levels of 0.9 mmol/L and total calcium levels of 1.7 mmol/L. Alfacalcidol and calcium carbonate were administered to the patient with normocalcemia achievement. In 2016, the target level of PTH (196.5 pg/mL) was recorded in combination with hypercalcemia (albumin-corrected calcium of 2.8 mmol/L). Therefore, the patient stopped taking vitamin D analogue and calcium carbonate.

Medical exams from 2016 until 2023 are not available. Etelcalcetide therapy began in 2022-2023 to treat progressive hypercalcemia. The dose was gradually increased to 15 mg/3 mL and administered three times a week, achieving a normocalcemia range of 2.2-2.3 mmol/L (based on total calcium).

M. was readmitted to the Endocrinology Research Centre in March 2023 at the age of 48 due to severe bone and joint pain in the hip, knee, and ankle, muscle weakness, decreased height, and partial hearing loss. There was no history of low-trauma non-spinal fractures. Menopause occurred naturally at the age of 47 years.

### Physical examination

**Figure 2:**
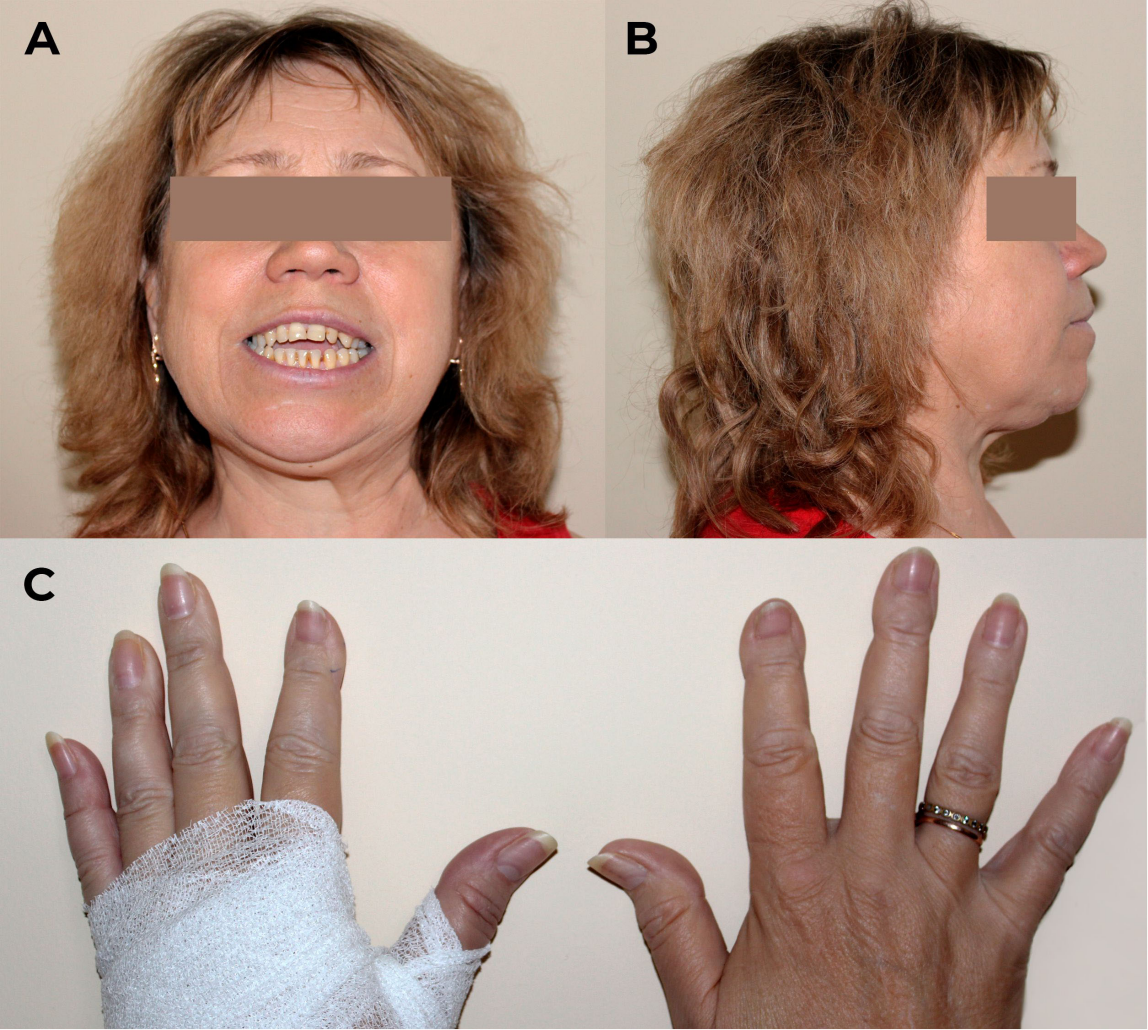
Physical examination of patient M. on admission. **(A, B)** Craniofacial anomalies affecting the maxilla and mandible, malocclusions, and dental irregularities. **(C)** Distal phalangeal deformities.

The physical examination revealed severe distal phalangeal deformities, altered posture, and a restricted range of motion in the hips and knees, as shown in **Figure 2**. The patient had a height loss of 5 cm in the last 10 years (from 174 cm to 169 cm) while her weight was 92 kg with body mass index of 32.2 kg/m^2^, indicating first-degree obesity. There were no other clinically significant features.

### Examination data and treatment

Laboratory tests were made on etelcalcetide therapy at 15 mg/3 mL three times per week. The PTH level of 1455 pg/mL (15–65) indicated recurrence despite an albumin-corrected calcium level of 2.2 mmol/L (2.15-2.55). Hyperphosphatemia of 2.4 mmol/L was also noted. Sevelamer hydrochloride was added to the regimen at 4800 mg three times daily. This resulted in a trend toward lower blood phosphate levels, which decreased to 1.9 mmol/L. The laboratory test results are shown in **Table 1**.

**Table 1:** Laboratory test results.

Laboratory test results (2023)		
Parameter	Results	Reference range
PTH, pg/mL	**1455**	15-65
Calcium (total), mmol/L	2,3	2,15-2,55
Albumin, g/L	44,4	30-50
Calcium (albumin-corrected), mmol/L	2,2	2,15-2,55
Phosphorus, mmol/L	**2,4**	0,74-1,52
Alkaline phosphatase (ALP), U/L	**179**	40-150
Osteocalcin, ng/mL	**300**	11-43
C-terminal telopeptide of collagen type 1, ng/mL	**6**	0,3-0,57
Dynamic changes in patient’s PTH levels		
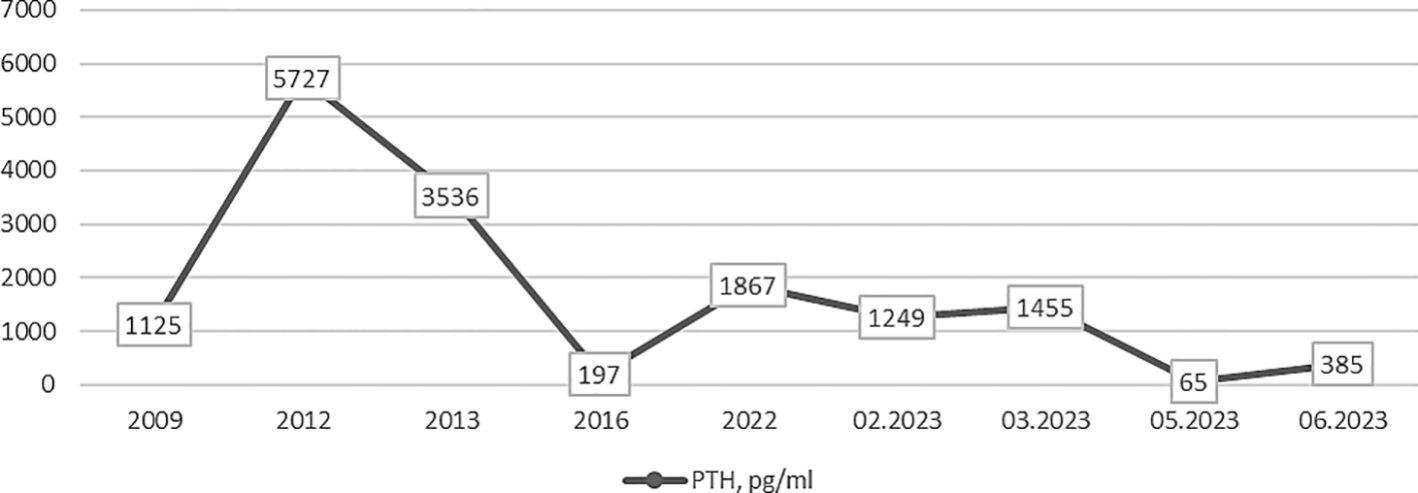		

**Figure 3:**
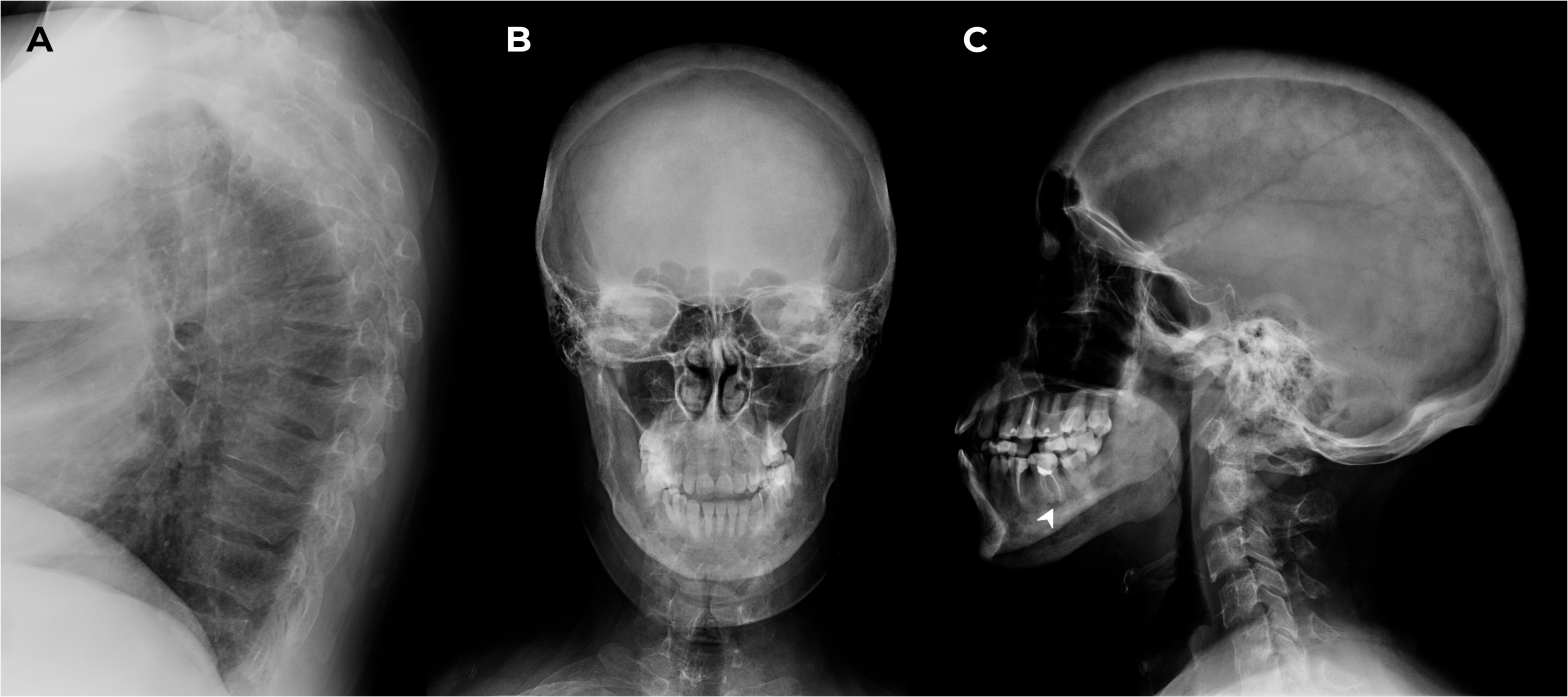
X-ray of the thoracic spine in lateral projection and the skull in frontal and lateral projections. **(A)** Th_6-8,12_ compression fractures. **(B, C)** Subcortical resorption on the alveolar masticatory surface (arrow) and multiple bone cysts in maxilla/mandible between 2 and 10.6 mm in the maxilla/mandible, multiple foci of fibrosis and endosellar calcification of the dura mater.

M. was screened for other mineral and bone disorders. Echocardiography demonstrated evidence of heart valvular calcification. Dual-energy X-ray absorptiometry scans (T-scores) showed severe reduction in bone mineral density in the femoral neck (-3.1 SD); less significant changes were in the lumbar spine and radius (-1.3 SD and -1.9 SD, respectively). The lateral radiographs of the thoracic and lumbar spine confirmed compression fractures at Th_6-8,12_ (28-58% vertebral body height loss) and initial decrease in vertebral height at Th_9-10_, L_1-5_ (10-18%) as shown in **Figure 3A**. These findings suggested negative progression compared to the 2016 assessment. Skull radiographs demonstrated pathological features typical for SS (**Figures 3B, C**).

Neck ultrasound identified a 3.8x2.9x2.3 cm nodule in the right lobe (EU-TIRADS 4). PTH levels in the washout fluid collected from a fine-needle aspiration (FNA) biopsy were greater than 5000 pg/mL. This finding confirmed the existence of the fifth intrathyroid PG.

In May 2023 the patient underwent a right-sided extrafascial hemithyroidectomy. On revision, the thyroid was enlarged at right lobe expense with a nodule up to 4.0 cm in diameter. The intrathyroidal PG was successfully excised, resulting in a beneficial outcome.

Macroscopically, the postoperative material was represented by the right thyroid lobe; at incision, it was mostly occupied by a soft elastic nodule of yellowish color with lobulated surface and a focus of decay surrounded by a capsule. The consistent histopathological and immunohistochemical findings confirmed multifocal PC pT2NxMx (**Figures 1B–F**).

PTH level decreased from 1366 pg/mL to 65 pg/mL within 60 minutes of surgery. There was no evidence of hypocalcemia in the early postoperative period.

### Follow-up

After surgery, M. was discharged from the hospital on alfacalcidol 1 mcg/day and colecalciferol 5000 IU/day therapy. Target PTH levels of 349.5 pg/mL (15–65) with albumin-corrected calcium of 2.2 mmol/L (2.15-2.55) were achieved on the therapy in August 2023. The patient notices an improvement of the condition, partial reduction of bone pain and fatigue.

## Discussion

SS is a rare manifestation of long-standing SHPT with inadequate compensation that develops in patients on programmed hemodialysis. The majority of cases described in the literature have been attributed to patients’ delayed access to appropriate treatment for SHPT due to low socioeconomic status and limited access to quality medical facilities (1).

M. was diagnosed with THPT, characterized by persistent hypercalcemia and markedly elevated PTH levels above 5000 pg/mL. However, she did not receive treatment for three years. In our case, the absence of medical intervention was explained by the patient’s circumstances.

As initial surgical treatment, the patient underwent total parathyroidectomy in 2013 to avoid the persistence or recurrence of the disease. Before surgery, pronounced bone deformities developed. However, during the period 2013-2023, there was no progression of skeletal abnormalities (except for negative dynamics of spinal compression fractures). Similar results have been shown by Ana Mejía Pineda et al. Total parathyroidectomy stopped musculoskeletal change progression in 5 patients with SS (observation period was restricted to only 14 months) (6). However, established deformities due to SS are not reversible and affect the patient’s quality of life (7).

### Etiology and pathogenetic associations

Severity of SHPT is characterized by the progression of hypocalcemia, hyperphosphatemia, elevated levels of intact PTH, and alkaline phosphatase due to impaired renal activation of 25-hydroxyvitamin D and hypersecretion of FGF-23 in patients receiving programmed hemodialysis. These findings do not explain the occurrence of SS in a specific subgroup of patients. The study by Ismail Yildiz et al. reported that there was no connection between the levels of 25-hydroxyvitamin D, calcitonin, somatotropic, thyrotropic, and gonadotropic hormones, and testosterone with bone deformities development (2). Various factors such as gender, age, duration of dialysis, and high levels of serum ALP may be considered as potential risk factors for SS (8, 9).

Patients with SS and their first-degree relatives did not show any significant chromosomal abnormalities or mutations in exons 2 and 3 of the *CASR* gene. Nevertheless, a subset of patients exhibited multiple missense mutations in the *GNAS1* gene, as well as in the *FGF23* and *FGFR3* genes, thus genetic associations may have a role in the onset of the disease (2, 10). Another study reported a statistically significant increase in the number of chromosomal aberrations in patients with SS compared to the control group (11). Further research is needed to clarify the exact etiology and pathogenesis of SS in patients with SHPT.

Previous case reports indicate that SS primarily occurs in young patients, with an average age of 30 [26; 37] years. Symptom severity, especially skeletal deformities, appears to correlate with the duration of hemodialysis (on average 7 [6; 13.5] years after the start), in the absence of adequate compensation for SHPT. PTH levels were significantly elevated, with a mean of 2686 [1286; 3624] pg/mL (9, 12–25). The presented data correspond to our patient’s findings. At the time of skull and thoracic deformities, M. was 38 years old, and the duration of programmed hemodialysis was 5 years with a maximum increase in PTH levels up to 5727 pg/mL. It appears that the relatively short period of hemodialysis was compensated by the aggressiveness of the mineral and bone metabolism disorders in SHPT, leading to the development of the SS phenotype. Unlike all cases describing facial skeletal changes, finger deformities are reported in about one in two cases (12–15, 17, 19–23). During the examination, we noticed thickening and slight concavity of the distal phalanges of the patient’s fingers.

Systemic hypocalcemia in severe SHPT triggers PTH-mediated osteoclast activation, resulting in brown tumors. They can be found in SS, but they are not an obligatory feature. Usually, these tumors occur as either unifocal or multifocal growths and predominantly affect the maxillary region (26).

Alper N. Erkan and colleagues found that approximately 60% of SS patients experience hearing loss (27). Our patient also presented with partial hearing loss. Her medical history revealed no previous trauma, diagnosed otolaryngologic conditions, or use of ototoxic medications.

Patients with SS frequently display diverse neuropsychiatric symptoms, such as headaches, insomnia, irritability, increased anxiety, depressive states, and various forms of paresthesias. Prolonged hemodialysis may contribute, in part, to these symptoms (3, 28). M. did not receive a comprehensive neurological or psychiatric examination. She never had problems communicating with medical staff. However, some emotional lability and increased anxiety were observed.

There are usually two pairs of PGs. In some cases, the number of these additional glands can reach up to 12. It is important to note that the intrathyroidal location of PC is extremely rare, with only about 20 documented cases reported in the literature (29, 30).

According to the 2022 WHO Classification of Parathyroid Tumors, in the absence of metastatic lesions, the «gold standard» for PC diagnosis remains evidence of tumor invasion into blood or lymphatic vessels, nerves, and adjacent anatomical structures. Tissue staining for parafibromin is an additional method that expands the diagnostic possibilities in the evaluation of parathyroid tumors (PT). In the case of complete loss of parafibromin, the PT is referred to as «parafibromin-deficient» and requires genetic testing for a *CDC73* gene mutation to differentiate between sporadic and genetically determined PC (31, 32). In the case we presented, PC was diagnosed based on vascular invasion. Given the complete loss of parafibromin on immunohistochemistry, genetic follow-up was recommended but unfortunately not performed.

A total of 37 cases of PC associated with SHPT or THPT have been reported in English-language literature. Recent reports have suggested a possible relationship between long-term SHPT and malignant parathyroid cell transformation due to their hyperstimulation (33). Notably, nearly 3% of PCs were found in patients on hemodialysis (34). In addition, laboratory changes in hemodialysis patients may overlap with the clinical picture of PC, further complicating the diagnosis (35, 36). Since our patient had no obvious risk factors for PC (no exposure to radiation and no family history of cancer), one of the probable factors in the development of PC in this case could be the patient’s prolonged stay on hemodialysis without hyperparathyroidism compensation.

The combination of recurrent THPT due to intrathyroidal PC in the same individual represents an exceedingly unusual and distinctive case. We did not suspect the presence of PC in our patient based on the preoperative examination findings. However, the finding of a solitary thyroid mass on ultrasound was suspicious for intrathyroidal PG. To investigate further, we punctured the thyroid mass and analyzed the levels of PTH in the needle wash.

Examination of FNA material to differentiate between thyroid and parathyroid tumors can be extremely difficult and is usually inappropriate (37). However, if intrathyroid PG is suspected, FNA followed by measurement of PTH in needle washout is a useful tool to verify the parathyroid origin of the tumor. In the case of preoperative suspicion of PC, FNA is not performed due to the risk of local metastatic spread of the tumor (38). Although this condition is extremely rare, it should be considered when ordering FNA (39–41).

## Conclusion

SS is a rare condition that develops in individuals with end-stage CKD and long-term, uncontrolled SHPT. This syndrome is characterized by significant skeletal deformities, particularly of the cranial bones, as well as other severe impairments disrupting the quality of life. Delayed diagnosis and inadequate treatment of SHPT may contribute to its development. Parathyroidectomy can stop the progression of the disease, but the existing bone deformities cannot be reversed. It is crucial to recognize, diagnose, and manage bone and mineral disorders in patients receiving renal replacement therapy, as well as to monitor these patients for possible SS development.

## Data availability statement

The original contributions presented in the study are included in the article/supplementary material. Further inquiries can be directed to the corresponding author.

## Ethics statement

Written informed consent was obtained from the individual(s) for the publication of any potentially identifiable images or data included in this article.

## Author contributions

RS: Conceptualization, Visualization, Writing – original draft, Writing – review & editing. EVB: Investigation, Visualization, Writing – review & editing. AE: Data curation, Investigation, Supervision, Writing – review & editing. EB: Data curation, Writing – review & editing. EK: Writing – review & editing. KB: Writing – review & editing. IK: Data curation, Writing – review & editing. SK: Writing – review & editing. NM: Data curation, Supervision, Writing – review & editing.
